# Asthma insights and reality in the United Arab Emirates

**DOI:** 10.4103/1817-1737.69109

**Published:** 2010

**Authors:** Bassam Hassan Saleh Hassan Mahboub, Sonia Santhakumar, Joan B. Soriano, Ruby Pawankar

**Affiliations:** *Department of Pulmonology, Rashid Hospital, Dubai, P.O. Box 4545, United Arab Emirates*; 1 *Program of Epidemiology and Clinical Research, CIMERA, IIIes Ballears, Spain*; 2 *Department of Allergy, Nippon Medical School, Tokyo, Japan*

**Keywords:** Asthma, burden, Gulf and near east, population, UAE, under treatment

## Abstract

**BACKGROUND:**

The burden of asthma in the United Arab Emirates (UAE) and the extent to which guidelines are being followed for optimum asthma control are largely unknown. This survey assessed the current level of asthma control, the burden of the disease, and adherence to asthma guidelines by patients.

**METHODS:**

A face-to-face interview of 200 asthmatics in the UAE was conducted. In addition to the questionnaire administered by expert interviewers, each respondent self-completed an Asthma Control Test. The sample was stratified by region within the country and sampled proportionately.

**RESULTS:**

Sudden severe attacks of asthma were reported by 64% in the past year. Day time symptoms and night time symptoms were reported by 57.5% and 35.5%, respectively, in the past 4 weeks. Overall, 52.8% of the children and 17.1% of the adults missed school and work in the past year, respectively. The percentage of asthmatics that had emergency room visits within the past year was 27.5%, and 4% were hospitalized. Only 5.5% used inhaled corticosteroids in the past year and 47.5% were on short-acting beta-2 agonists. Only 17.8% ever owned a peak flow meter and only 30% ever had a lung function test. Only 17% had scheduled follow-up and 66% were followed-up by general practitioners.

**CONCLUSION:**

This survey shows that the current level of asthma control in the UAE is far from optimal. Therefore, it is necessary to increase the awareness among patients and update doctors about asthma control guidelines for attaining optimal asthma control, and thus reducing the burden of the disease.

Asthma is a chronic inflammatory disease of the airways affecting more than 300 million[1] people worldwide. It can cause considerable burden on the physical, social, emotional, and professional lives of affected individuals and family.

The International Study of Asthma and Allergies in Childhood (ISAAC) and the European Community Respiratory Health Survey (ECRHS) have shown that there is an increase[2] in the global prevalence of asthma, particularly in children. Although the prevalence of asthma is more in the developed countries,[3] evidence in recent years, has shown a sharp increase in the developing countries, possibly due to the effects of urbanization.

There are no reliable data on the prevalence of asthma in the UAE to date. But some small studies[4] on the prevalence of asthma among school children in the UAE have shown that the prevalence of disease among school children is 13.6%.

Asthma when inadequately controlled[5] can affect the quality of life considerably, hence control of disease is mandatory. The Global Initiative for asthma was initiated[6] in 1989, in collaboration with The National Heart, Lung, and Blood Institute in 1993 to set up guidelines for the management and treatment of asthma. The last updated GINA in 2008[1] has focused on staging of asthma according to disease control, rather than staging based on its severity.

The Asthma Insights and Reality (AIR) surveys are part of comprehensive multinational surveys conducted in adults and children worldwide to assess the current level of asthma control among asthmatics. Similar surveys have been conducted and reported in Europe (AIRE),[7] Asia pacific,[8] America,[9] Japan,[10] and Latin America.[11] All of them highlighted that asthma is still globally undertreated and adherence to asthma guidelines is far from optimal.

The AIR survey in the UAE is part of The AIR survey in the Gulf and Near East (AIRGNE), which has also included Jordan, Lebanon, Kuwait, and Oman. AIRGNE is the first and largest comprehensive study done in the region to date, which assessed the level of control of asthma and adherence to treatment guidelines.

## Methods

### Selection of subjects

This survey was conducted between January 2007 and March 2008.The total population in the UAE is about 2,563,000 of which only 23% are UAE nationals. The most populated urban areas in the UAE, namely, Abu Dhabi, Dubai, and the Northern Emirates were included in the study.

Sampling was structured according to the gender and age within the city or region to provide a representative sample. The sampling quotas were made of nationals (50%), and nonnational groups. The nonnational group comprised other Arabs (25%), South Asians (65%), and East Asians (10%). Accordingly, structured quota samples were applied within cities by gender, age, and nationality, so each interviewer was allotted an area plus a set quota for that area.

### Sampling methods

Asthma patients in the country were identified by systematic surveying of households for persons who had been diagnosed as suffering from asthma. The sampling plan was designed to provide a nationally representative sample of households that could be screened to identify a community sample of current asthmatics in the country. Sampling and interviewing limitations required the sampling frame be restricted to urbanized areas within the country. As mentioned above, geographically stratified samples of households proportional to the population in quotas by age and gender were drawn within the four areas of the country. The survey design required a sample of 200 asthmatics from UAE. In each household, an adult was asked whether a physician had ever diagnosed any member of the household as suffering from asthma. If the answer was yes, the interviewer asked whether any of those individuals were currently taking medications for their asthma or had suffered an asthma attack or experienced asthma symptoms in the past one year. Information regarding the number of persons in the household and those who had been diagnosed with asthma and met the survey criteria for current asthma were collected to provide comparative prevalence. If more than one household member was qualified as having current asthma, the interviewer randomly selected one as the designated respondent. Only one respondent was interviewed per household because multiple interviews in the same household can cause a bias. If the selected respondent was 16 years or older, the interview was conducted with the patient; if he or she was 15 years or younger, the interview was conducted with the parent or guardian who was more knowledgeable about the child’s condition and treatment.

### Questionnaire

The core questionnaire used in previous AIR[12] studies in Europe and elsewhere, which was actually based on the original ATS[13] questionnaire, was used. The questionnaire was translated to Arabic and then translated back to English and any discrepancies and inconsistencies were solved by consensus. Finally, the self-completed Asthma Control Test[14] (ACT) and additional questions to reflect the local conditions and characteristics of asthma in the UAE and some questions to reflect the latest version of GINA guidelines were additionally included. The questionnaires were printed in English–Arabic side-by-side layout. As per quality control, all the materials were piloted and there was a personal briefing of the interviewers and each interviewer conducted two pilot interviews and reviewed two completed questionnaires with a supervisor before starting the field work. Completed questionnaires were checked locally by the Ministry of Saudi Arabia and again centrally by IMS (Central UK office) and there was a random back checking of interviews conducted by telephone. Finally all the data were included in a database after independent double typing.

### Data analysis

The data on asthma morbidity and management practices were stratified by age (children: 15 years or younger; adults: 16 years or older) because existing asthma treatment guidelines are different for children and adults and the responses for children were obtained by proxy. The frequency and severity of daytime and nighttime symptoms, exercise-induced symptoms; severe episodes, and total symptom frequency were used to determine asthma symptom severity. The events, such as hospitalization and utilization of emergency services as well as loss of school/work days due to asthma were documented. The patients’ demography and asthma severity were compared using Chi-square analysis to identify factors that might account for differences in asthma management across the country or analysis of variance for quantitative variables whenever required. Statistical comparisons (SPSS for Windows version 12.0) of reports within the country vs published international AIR reports were done. All statistical tests were two-sided and comparisons with <5% probability of error were considered statistically significant.

## Results

### Sample population

A total of 200 asthmatic subjects across UAE were surveyed in this study. Thirty-four percent of the total participants were from Abu Dhabi and 33% from Dubai, and the remaining 33% from the Northern Emirates. Eighteen percent were younger than 16 years and 82% were adults. Eighty-five percent of the asthmatics were males and 10% of the participants were current smokers and 6% exsmokers. The average age in years that is mean ± SD of the participants was 28.4 ± 14.5 years. The mean age at the time of diagnosis of asthma was 15.0 ± 11.9 years [Table 1].

**Table 1:** Demographic characteristics of asthma patients

Parameter	Population n = 200
Interval age distribution (years)	
5-15	36 (18)
16-29	60 (30)
30-49	86 (43)
> 50	18 (9)
Mean age at diagnosis (years)	15.0 ± 11.9
Mean age of participants (years)	28.4 (14.5)
Gender	
M	170 (85)
F	30 (15)
Level of education	
Primary	4 (2)
Secondary	52 (26)
University	140 (70)
Income	
< 12000 AED	48 (24)
12000-15000 AED	48 (24)
15000-25000 AED	52 (26)
>25000 AED	52 (26)
Region	
Abu Dabi	68 (34)
Dubai	66 (33)
Northern Emirates	66 (33)
Smoking *habits* in adults	
Current	20 (10)
Former	12 (6)
Never smoked	168 (84)

Values correspond to numbers (percentages) of patients in the corresponding category except for age at diagnosis, which are represented as mean ± SD

### Asthma control

**Figure 1:**
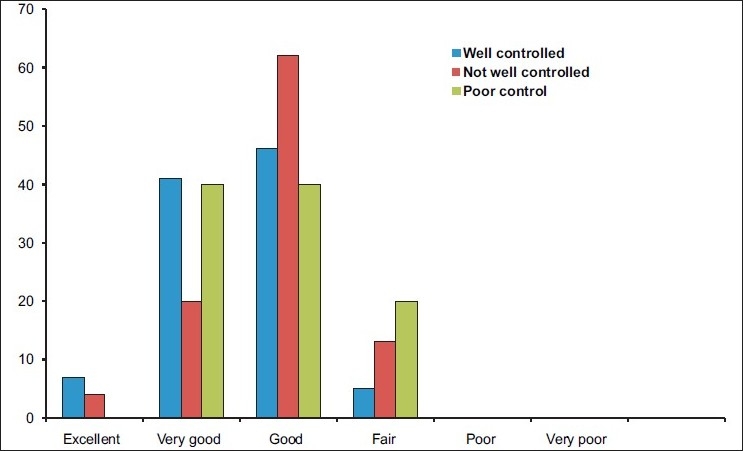
General health by asthma control based on ACT

Based on the ACT score, the majority of patients (70%) perceived their asthma was well controlled. The mean ± SD ACT score was 20.9 ± 3.2, although there was no good agreement with subjective asthma control (χ^2^ *P* value < 0.05) [Table 2] or with general health [Figure 1].

Over half of them believed their asthma was getting better (59%), and the majority (71%) reported seasonal asthma.

**Table 2:** Comparison between objective and subjective evaluation of asthma control

	Subjective asthma control	Objective asthma control	*P*
Poorly and not well controlled	8 (4)	60 (30)	<0.05
Well/completely controlled	192 (96)	140 (70)	<0.05

*Objective evaluation of asthma control according to the Asthma Control Test (ACT). An ACT of 5 to 19 corresponds to a poorly or not well controlled asthma, and an ACT of 20 to 25 corresponds to a well controlled asthma, Figures in parentheses are in percentage

### Morbidity due to asthma

There was substantial morbidity associated with asthma. Two thirds (64%) had suffered sudden severe episodes of asthma during the past year. About 40% had such attacks at least twice a year and 11% of children and 3% of adults had to be hospitalized for asthma. Twenty-one percent of the well-controlled group and 60% of the poorly controlled asthmatics had to utilize emergency resources during the past year. Sixty-one percent had at least some limitation of sport activities. Sixteen percent of adults and 53% of children had to miss their work or school during the past year. Daytime symptoms and nighttime symptoms in the past four weeks were reported by 57.5% and 35.5%, respectively. Of these, 30% had daytime symptoms and 27% had nighttime symptoms at least twice a week [Table 3].

**Table 3:** Evaluation of asthma burden in the past year in UAE by comparison to AIRGNE results

	UAE	AIRGNE total
Asthma burden in the past year		
School absence in children, %	53	51.7
Work absence in adults, %	16	29.7
Mean number of days	4.4	7.3 (8.1)
Use of health services in the past year		
Hospitalization, %	4	22.5
Emergency room visit, %	28	51.5

In comparison with all the AIRGNE participants, children in the UAE had similarly high lost school days during the past year (52%) (χ^2^ *P* value > 0.05). However, the adults reported a substantially lower burden compared with the AIRGNE participants in terms of work absence, number of lost days, emergency visits, and hospitalization (all χ^2^ *P* value < 0.05) [Table 3].

Less than half of the participants had ever heard of a peak flow meter and only 8% (n=16) had ever used it. Only 25% of the children and 37% of the adults had ever undergone a lung function test.

Only 31% were familiar with inhaled corticosteroids and only 5.5% were using them. Sixty-seven percent were using short-acting beta-2 agonist (SABA) as their current medication and most (57%) believed that SABA is the most effective drug for controlling asthma symptoms.

Almost 70% were diagnosed as having asthma and were being followed-up by general practitioners. Only 17% had scheduled follow-up visits and only 15% had a written action plan by the doctor, whereas just less than a quarter were aware of asthma guidelines.

**Figure 2:**
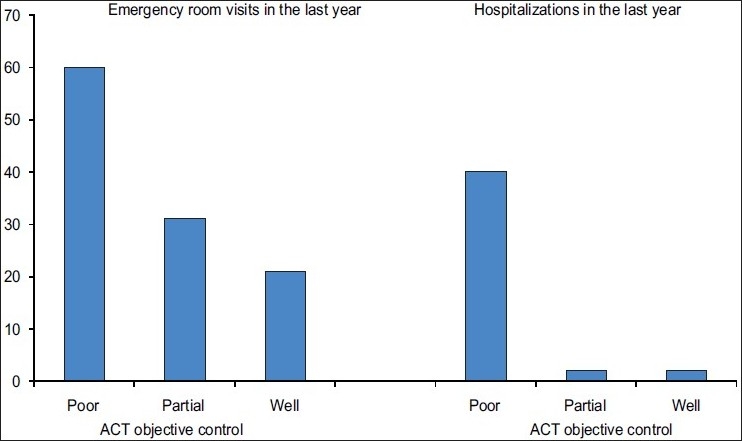
Percentage of emergency room visits and hospitalizations in the last 12 months according to the ACT score

Finally the ACT correlated well with increased utilization of health services, either emergency room visits (linear χ^2^ 4.448, *P* value=0.035) or hospitalization (linear χ^2^ 12.009, *P* value= .001) [Figure 2].

## Discussion

The AIR UAE study, which was conducted as part of the AIR GNE (first study in the region), was the first survey in the UAE that assessed the current level of asthma control and management. As in other studies of the kind conducted worldwide, these surveys provide direct evidence that despite the availability of guidelines and effective medication the burden of asthma in the UAE is unacceptably high. The majority of patients had acute attacks and 40% had unscheduled emergency visits. The disparity between the patient’s perceived level of asthma control and GINA goals of asthma control is striking. Although 89% of asthmatics felt their general health was at least good, most of them had chronic symptoms and many had limitation of activities and loss of school/work days. High reliance on controller medication and significant underuse of preventive medication is another disturbing finding. These findings show that majority of asthmatics in the UAE are undertreated and the GINA goals of treatment of asthma are not yet met.

Data on asthma prevalence and morbidity in the UAE are scarce. A cross-sectional study on 850 school children conducted by Bener *et al*.,[4] in 1994, showed that the prevalence of asthma among the school children was 13.6%. In 2000, another study was conducted among school children in the UAE by Al-maskari and colleagues. The ISAAC study was conducted in Kuwait,[15] Morocco, Oman, and Lebanon but not in the UAE.

### Limitations of the study

Random digit dialing (RDD)[16] was used in many previous AIR surveys. In countries where telephone coverage is close to 100%, RDD can approximate a representative random sample of the population. However, RDD was not considered in this survey because of social norms, low penetration of telephone coverage, and lack of availability of telephone number databases. The urban sampling frame used in this study would have created a selection bias toward higher socioeconomic groups rather than the country as a whole. In the absence of comprehensive demographic information, it is hard to assess the magnitude of the bias. However, this does not influence the overall conclusions reached from this survey, as it points to lack of sufficient awareness as well as undertreatment of asthma. One would presume that in the rural population, the results would have been worse[17,18] or more or less the same because of economic constraints and reduced availability of health care facilities.

One of the regional factors that are worth considering is the problem associated with the term ″asthma″ in Arabic, which means acute severe problem, and hence many doctors use the term allergy making it more acceptable to patients. There is also no good translation of ″inflammation″ in Arabic. This might have caused the patients to underestimate their disease severity and to rely on rescue medication for the treatment of their condition.

As with other AIR surveys,[6] our study also highlights the discrepancies between patients’ perceived asthma control and symptom severity. Patients adapt themselves to their condition and overestimate their control, thus settling for a quality of life much less than that is achievable if management guidelines are followed. This is evident from the fact that, although 30% of the participants had poorly controlled or uncontrolled asthma when measured objectively using ACT, only 8% were aware of it. Only 17% believed that their condition could be treated. Increasing public awareness of the disease and improving both patient’s and physician’s expectations can go a long way in improving overall asthma control.

Our survey is in conformity with the earlier surveys of the kind, concerning the underutilization of inhaled corticosteroids (ICS). It may be noted that only 5.5% of the participants were using ICS, which was much less when compared with the overall AIRGNE results of 14.5%. Only one third of them were familiar with ICS and the majority believed that the risks possibly outweighed the benefits. This calls for better communication between patients and physicians[19] and patient education regarding the benefits and the imperative need for the use of preventive medication for long-term management of asthma.

The GINA goal of monitoring and long-term management of asthma are also unmet as can be seen from the results of the survey. Only 30% of the participants had a lung function[20] test. Only 8% had ever used a peak flow meter. The majority did not have a written action plan for asthma control and only 17% had scheduled follow-up visits.

In conclusion The AIR in UAE survey indicates that asthma in the UAE is undertreated and adherence to asthma guidelines is poor. It is imperative that general physicians who are responsible for the diagnosis and management of most asthmatics need to be updated to improve the asthma control methods and to lessen the gap between long-term goals of asthma management and the existing condition in the UAE.
